# S1PR1 and VEGFR2 – a synergy that promotes tumor angiogenesis?

**DOI:** 10.1080/23723556.2020.1746131

**Published:** 2020-04-07

**Authors:** Vijay Avin Balaji Ragunathrao, Vigneshwaran Vellingiri, Mumtaz Anwar, Md Zahid Akhter, Dolly Mehta

**Affiliations:** Department of Pharmacology and the Center for Lung and Vascular Biology, University of Illinois College of Medicine, Chicago, IL, USA

**Keywords:** S1PR1, VEGFR2, cAbl1, tumor angiogenesis

## Abstract

We have recently uncovered that endothelial cell (EC) S1PR1 controls the effectiveness of VEGFR2 driven tumor angiogenesis. By using tumor ECs, EC-S1PR1^−/-^ mice and S1PR1 antagonist, we showed that VEGF-VEGFR2 pathway requires EC-S1PR1-induced signaling to efficiently drive tumor vascularization and growth, indicating combining S1PR1 antagonist with anti-VEGF/VEGFR2 therapy may eradicate resistant tumors.

## Authors commentary

Assertive vascularization of tumors is a crucial factor responsible for tumor growth and metastasis which is mediated by VEGF-VEGFR2 (Vascular endothelial growth factor-VEGF receptor 2) signaling.^1^ However, treatment with VEGF and VEFGR2 inhibitors has only been efficacious in some cancers and further it has been largely ineffective in preventing metastasis and recurrence of tumors.^2^ Studies show that other growth factors intersects with VEGFR2 signaling which may dictate the outcome of VEGFR2 angiogenic signaling.^3^ In this context, we surmised that other angiogenic pathways co-operates with VEGF-VEGFR2 signaling to efficiently drive VEGFR2 tumor vascularization and growth.

Sphingosine-1-phosphate receptor 1 (S1PR1) expressed on EC-surface, like VEGFR2, is shown to mediate tumor angiogenesis.^4^ Upon ligating sphingosine 1 Phosphate (S1P), S1PR1 stimulates heterotrimeric protein, Gi which in turn activates PI3-kinase/Akt/ERK signaling leading to EC survival.^5^ S1PR1-Gi cascade also activates the small GTPase Rac1, which is essential for EC migration.^6,7^ While a few studies showed that impairment of S1PR1 function reduced VEGF-induced angiogenesis^8^ others showed that loss of S1PR1 in ECs induced hyper-sprouting due to increased VEGFR2 activity.^9^

**Figure 1.**
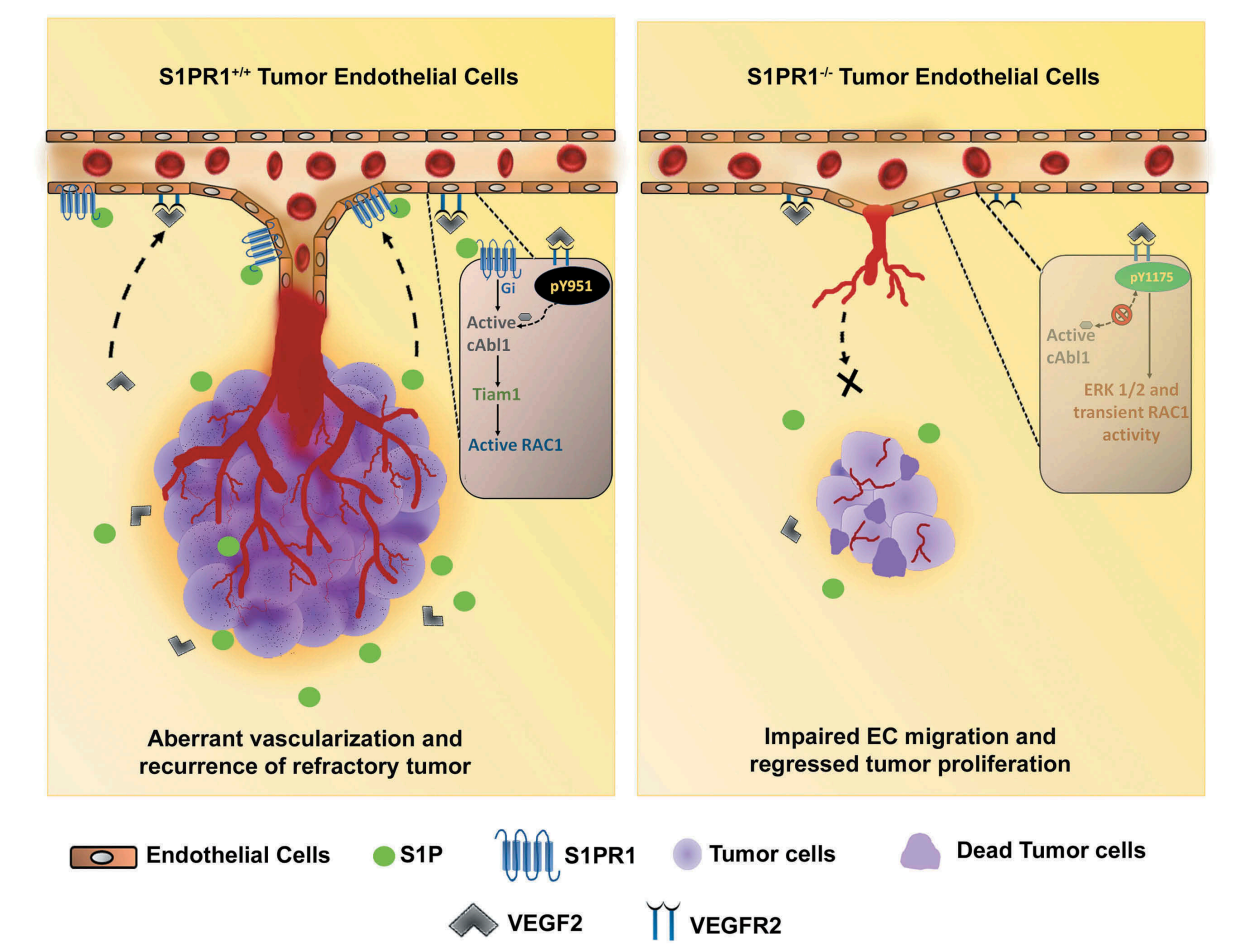
Mechanisms whereby VEGFR2 and S1PR1 promote tumor growth. VEGF/VEGFR2 and S1P/S1PR1 signaling synergistically regulates endothelial migration and thereby tumor angiogenesis. S1P generated from cancer cells ligates S1PR1 in S1PR1 + ECs, stimulates Gi, which in turn promotes c-Abl1 activity by VEGF leading to the VEGFR2 phosphorylation at Y951 results in persistent activation of VEGFR2 on EC plasmalemma. The relentless synergistic activity of VEGFR2 and S1PR1 in ECs prolongs Rac1 activity in a Tiam1 dependent manner leading to the establishment of tumor vasculature and have an advantage for recurrence of aggressive refractory tumor after anti-VEGFR2 therapy. While, in ECs lacking S1PR1, VEGF activated VEGFR2 pursues canonical phosphorylation at Y1175 followed by receptor internalization to activate ERK1/2/3 leading to impaired EC migration and tumor vascularization.

We demonstrated that endothelial S1PR1-cAbl1 signaling pathway prolonged VEGFR2 signaling augmenting thereby tumor angiogenesis and growth.^10^ While S1P and VEGF can both independently mediate angiogenesis through activation of their respective receptors, S1PR1 and VEGFR2 in ECs.^4^ We showed that VEGF alone was weakly angiogenic in S1PR1-depleted ECs corroborating the study shown by LaMontagne *et al*^8^ indicating that, that S1PR1 was required for promoting VEGFR2 mediated ECs migration and angiogenesis. The importance of SIPR1/VEGFR2 interaction was evident in studies showing conditional deletion of S1PR1 in ECs markedly reduced VEGFR2 mediated tumor angiogenesis.^10^ These findings are in line with previous published studies, for example, S1PR1 facilitated VEGFR2 mediated migration of thyroid cancer cells^4^ while blockade of S1P using anti-S1P antibodies or S1PR1 antagonists prevented VEGF-induced tumor angiogenesis and growth.^8,11^ Thus, our study demonstrated that EC-S1PR1 controlled the efficiency of VEGFR2 signaling to drive the key tumor angiogenesis program, i.e. EC migration supporting tumor vessel vascularization and growth (Figure 1). However, a caveat of the Vijay Avin *et al* study was that animal model used did not allow an assessment of the role of SIPR1 enhancement of VEGFR2 angiogenic signaling in promoting tumor resistance to VEGFR2 based therapy which represent an attractive topic for future investigations.

The current dogma regarding VEGFR2 angiogenic signaling is that upon ligating VEGF the receptor is phosphorylated at tyrosine (Y) 1175.^3^ Y1175-phosphorylated VEGFR2 then internalizes and induces the activities of small GTPase Rac1, ERK and AKT for signaling angiogenesis.^3^ Thus, in contrast to S1PR1, internalized VEGFR2 drives angiogenesis. Additionally, a few studies showed that loss of VEGFR2 phosphorylation at Y951 residues (949 in mouse) blocked tumor metastasis.^12^ Vijay Avin *et al* demonstrated that S1PR1 inhibited VEGFR2 internalization by VEGF and in this case VEGF phosphorylated VEGFR2 at Y951. Cell-surface retained VEGFR2 persistently activated Rac1 following VEGF addition which in turn promoted EC migration to tumor for vascularization and growth. However, in the case of S1PR1 null tumor ECs, VEGF resulted activation of the canonical pathway in which VEGF induced phosphorylation of VEGFR2 at Y1175 was followed by internalization with the co-operative endocytic machinery leading to activation of ERK1/2/3.

S1PR1 couples with heterotrimeric GTP binding protein, Gi to induce downstream signaling.^5^ Using pertussis toxin which blocks Gi activity we showed that S1PR1 required Gi activity to control VEGFR2 phosphorylation at Y951. The tyrosine kinases, c-Src, FAK, and c-Abl can phosphorylate VEGFR2.^3^ We demonstrated that cAbl1, but not c-Src or FAK phosphorylated VEGFR2 at Y951 downstream of S1PR1 and Gi. c-Abl1 activity is known to induce tumors,^13^ further highlighting the relevance of our findings.

The kinase activity of c-Abl1 is increased following binding to its substrate.^13^ In order to investigate whether S1PR1 mediated c-Abl activity by complexing it with Gi we performed co-immunoprecipitation assay. We demonstrated that VEGFR2 interacted with cAbl1, and further that Gi was required for cAbl1 activation downstream of S1PR1. Moreover, siRNA-mediated knockdown of cAbl1 prevented VEGFR2 phosphorylation at Y951 as well as Rac1 activation. Expression of Y951-phosphodefective VEGFR2 mutant also reduced cAbl1 phosphorylation in response to VEGF, suggesting an amplification mechanism of cAbl1 activation. In fact, neuropilin 1 receptor interacts with VEGFR2 by a mechanism involving cAbl1 phosphorylation of VEGFR2 at Y951 supporting the findings of the current study.^14^

Taken together, we showed that the VEGF-VEGFR2 pathway requires S1PR1-induced signaling in ECs to efficiently drive tumor vascularization and growth. S1PR1 functions via Gi to promote the activation of cAbl1 by VEGF. Activated cAbl1 induces phosphorylation of VEGFR2 at Y951, promoting receptor retention at the EC surface which in a sustained manner induces Rac1 activity and EC migration and tumor angiogenesis (Figure 1). Results from the present study suggest that a combination of S1PR1 antagonist with VEGF-VEGFR2 inhibitors could represent the first step toward treating VEGFR2 refractory tumors and prove relevant in other cancers driven by VEGF-VEGFR2 signaling.
